# Longitudinal predictive validity of emotional intelligence on first year medical students perceived stress

**DOI:** 10.1186/s12909-017-0979-z

**Published:** 2017-08-18

**Authors:** Richa Gupta, Nikhilesh Singh, Ramya Kumar

**Affiliations:** 1 Department of Physiology, Mahatma Gandhi Medical College and Research Institute, Sri Balaji Vidyapeeth University, Pondicherry, 607402 India; 2 Department of Physiology, Government Villupuram Medical College, Villupuram, India

**Keywords:** Emotional intelligence, Perceived stress, Academic performance, Predictive ability

## Abstract

**Background:**

Emotional intelligence has been shown to affect academic performance and perceived stress. But conflicting reports suggest that the relationship between academic performance and emotional intelligence may not be straightforward. Hence, this study explored the relationship between emotional intelligence, perceived stress and academic performance.

**Methods:**

First year medical students were invited to participate in this longitudinal study. At Time 1, before mid-semester examinations, they completed the questionnaires on Schutte’s Emotional Intelligence Scale (SEIS) and Perceived Stress Scale (PSS) (*n* = 213). At Time 2, before pre university examinations, students again completed perceived stress scale questionnaire. (*n* = 138). Academic performance was reported using summative assessment at both T1 and T2. The relationship between academic performance, emotional intelligence and perceived stress was explored using regression analysis.

**Results:**

Neither PSS nor SEIS were related to academic performance. However, perceived stress was significantly predicted by SEIS both at T1 (*r* = 0.333, β = 0.149, *p* < 0.001) as well as T2 (*r* = 0.240, β = 0.116, *p* = 0.028). The results were cross-validated at student level both at T1 and at T2.

**Conclusion:**

Medical students with higher trait emotional intelligence perceived lesser stress. Therefore, it might be prudent to train medical students to increase their emotional intelligence to promote their well-being.

## Background

Emotional Intelligence (EI) is defined as “the ability to perceive emotions, to access and generate emotions so as to assist thought, to understand emotions and emotional meanings, and to reflectively regulate emotions so as to promote both better emotional and intellectual growth” [1]. Studies have reported that students with higher emotional intelligence report lower perceived stress [2, 3]. Hence, one’s ability to handle stressful factors affects perceived stress apart from large number of stressful factors and their quality [4].

Further, perceived stress has been shown to negatively correlate with both cognitive [5] and behavioral [6] aspects of academic performance. Additionally, same perceived stress might lead to different emotional responses, in turn, affecting the eventual cognitive and psychomotor responses [7]. This has been described as “trinity of stress” where emotional responses to stress govern the cognitive performance [8]. This suggests that emotional intelligence may play a role directly or indirectly on academic performance. Indeed, it has been suggested that cognition and emotion are inextricably interwoven in complex decision making tasks [9], which range from conflict detection to conflict resolution. However, there are conflicting reports of effects of emotional intelligence on academic performance in existing literature.

The claim is supported by studies that found that EI contributes to cognitive based performance independent of general intelligence [10]. Studies have revealed a positive correlation of EI and cognitive performance [11], interpersonal communication skills [12], and patient satisfaction [13]. In other studies, EI does not impact academic performance [14, 15]. Additionally, studies reporting the positive association of emotional intelligence with academic performance and of emotional intelligence with perceived stress conspicuously lacked cross validation, which questions their predictive validity. Despite the lack of such literature, there has been widespread call of training the health care personnel in improving emotional intelligence [16].

Existing conflicting reports of the effect of EI on academic performance, suggest that the relationship between EI and academic performance may not be straightforward [17]. A recent study has explored the role of emotional intelligence as a link between perceived stress and subjective depression and happiness scores [18], but the link has not been studied for objective measure of performance and is worth exploring. It will not only help in devising strategies for curricular development but also provide an evidence based framework for improving medical students’ well being as there exists high prevalence of stress in first year medical students [19] and their well-being has shown to be negatively affected by high level of perceived stress [20].

In light of conflicting reports and lack of literature on predictive validity, we have focused on exploring the effect of emotional intelligence and perceived stress on academic performance in this paper.

## Methods

### Ethics, consent and permissions

This prospective cross-sectional study was done in a private medical college in Pondicherry from December 2014 to June 2015. Institutional ethics committee at Mahatma Gandhi Medical College and Research Institute, Pondicherry approved the study. All participants consented to be part of the study.

### Participants

First year MBBS students of 2014–15 batch were invited on voluntary basis 1 week before midterm examinations (T1) to participate in the study. They were informed that no harm would happen to them for non-participation and they can leave the study at any time. Students were again approached after 5 months, before pre-university exams (T2), to participate in the follow-up of the study.

### Methods

At T1, students received information sheet, consent forms along with paper and pencil Schutte’s Emotional Intelligence Scale (SEIS) and Perceived Stress Scale (PSS) questionnaire. It took them approximately 30 min to complete both the questionnaires in private. PSS questionnaire was given again to the students at T2.

Academic performance was reported using summative assessment scores of their physiology mid semester marks at T1 and pre university marks at T2. Mid semester written examination composed of 2 h 30 min written examination consisting of; one essay question (10 marks), 6 short answer questions (4 marks each), 8 very short answer questions (2 marks each) (Total of 50 marks). Pre-university examination at T2 composed of 2 consecutive day written examination on the same pattern as mid-semester examination (Totalling 100 marks of two papers). To maintain the uniformity of assessment, same faculty corrected same questions for all students.

The study design required the students to provide their names or Id’s for comparison of their responses between T1 and T2 and their academic performance. As involved researchers were of 1st year faculty, on ethical grounds, the responses of the participants were placed in sealed envelopes at T1 as well as at T2. These envelopes were made available to the research team only after students passing their 1st year.

### Rating scales

Schutte’s Emotional Intelligence Scale (SEIS) is a well established self report instrument to measure trait emotional intelligence [21]. It comprises of 33 items, 3 of which are reverse scored. Participants respond on a 5 point Likert scale ranging from strongly disagree (=1) to strongly agree (=5). Total EI score is calculated by summing the responses. Minimum EI score is 33 and maximum is 165. Stress was measured by perceived stress scale (PSS) comprising of 10 items, 4 of which are reverse scored [22]. The scale measures the stress perceived by the participants in the previous month. Total score is calculated by summing the responses. Minimum and maximum perceived stress scores are 0 and 40 respectively.

### Statistical analysis

Data was analyzed using SPSS version 16. Tests of significance were two tailed, and a *p* value of less than 0.05 was considered as statistically significant.

## Results

In total, 213 responses were obtained during T1 and 138 during T2. Eleven responses from T1 and 3 responses from T2 were excluded from the study as response to questionnaires had more than one missing value. In effect, 202 responses from T1 and 135 responses from T2 were used for further analysis. Sample size was sufficient to detect R square greater the 0.12 with 80% power at 5%level of significance both at T1 and T2 [23].

3.96% of responses at T1 and none at T2 had one missing value. Missing items for both SEIS and PSS were replaced by mean values. Cronbach’s alpha of SEIS was 0.88 and of PSS was 0.81 at T1 and 0.83 at T2. Both the scales were found to be internally reliable for further analysis. Studies have provided different and controversial factor solutions for SEIS. Hence, it has been emphasized previously that scale should be factor analyzed before using it [24]. Since, we did not have any firm idea about number of factors which exist for SEIS, we performed exploratory factor analysis to decide upon which scores to use for further analysis.

Factor analysis of responses of SEIS showed all questions, except question number 33, loaded on a single factor. Therefore, that question was removed from further analysis and total EI score was calculated by summing up rest of the responses.

Data was explored for outliers by Mahalanobis distance and analyzed for assumptions of multivariate analysis. Four outliers from T1 (*n* = 198) and three from T2 (*n* = 132) were removed. Descriptive statistics and demography of the sample is provided in Tables 1 and 2.

**Table 1:** Mean (standard deviation) of age, emotional intelligence score (EI), perceived stress score (PSS1) and mid semester marks at T1

	(*n* = 198)	Male (*n* = 107)	Female(*n* = 91)	*p* value
Age	18.81(0.64)	18.78(0.69)	18.84(0.582)	0.18
EI	118.97(14.05)	118.10(13.86)	120.0(14.28)	0.32
PSS 1	19.46(6.15)	18.88(5.75)	20.14(6.56)	0.15
Acad 1	36.19(11.92)	34.10(12.43)	38.63(10.86)	0.0076*

*Statistically significant

**Table 2:** Mean (standard deviation) of age, emotional intelligence score (EI), perceived stress score at T2 (PSS2), perceived stress score of matched students at T1 (PSS1) and pre-university marks at T2

	(*n* = 132)	Male (*n* = 69)	Female (*n* = 63)	*p* value
Age	18.83 ± (0.93)	18.90 ± 1.16	18.76 ± 0.59	0.25
EI	120.47(12.36)	120.17(12.65)	120.79(12.12)	0.77
PSS 1	19.21 (6.09)	18.97(5.86)	19.48(6.36)	0.64
PSS 2	18.25(5.77)	17.25(6.59)	19.35(4.52)	0.003*
Acad_2	58.70(21.94)	52.87(22.20)	65.19(19.87)	0.001*

*Statistically significant

Dropout analysis did not reveal any difference in SEIS scores, PSS scores and academic performance of dropouts (T1) when compared with other respondents (T1). Results are provided in Table 3. Further analysis was done only on the data of participants who completed the questionnaire at T1 as well as T2.

**Table 3:** Comparison of emotional intelligence (EI), perceived stress score (PSS1) and academic performance (Acad1) of dropouts and other responders at T1

	Dropout (*n* = 66)	Responders (*n* = 132)	*P* value
EI	117.17 ± 15.00	120.47 ± 12.36	0.101
PSS	19.96 ± 6.30	19.21 ± 6.09	0.425
Acad 1	35.21 ± 11.55	36.76 ± 12.09	0.391

Paired t-test for perceived stress score at T1 and T2 (*n* = 132) showed perceived stress at T2 was marginally less than at T1 (*p* = 0.065). On exploring for gender difference using paired t-test, there was no change in PSS scores for females (*p* = 0.856), but PSS scores of males was significantly less at T2 as compared to T1 (*p* = 0.024).

As PSS score, both at T1 and at T2, was not linearly related with academic performance, it was not considered for further analysis.

The data at T1 and T2 was randomly split into training (70%) and test (30%) data set. To explore the effect of emotional intelligence on objective performance, academic performance was regressed stepwise on EI scores and gender. The relationship was not found to be significant, given in Table 4. The effect of EI scores and PSS (T1) on academic performance (T2) was also analyzed by stepwise linear regression after controlling for gender and academic performance (T1). The effect was not found to be significant. To further explore the effect of emotional intelligence on academic performance, highest and lowest quartile data based on perceived stress were compared for difference in emotional intelligence and academic performance. There was no difference in academic performance of highest and lowest quartiles at T1 as well as T2. Results are provided in Table 5.

**Table 4:** Stepwise linear regression analysis evaluating the effect of emotional intelligence (EI) on perceived stress score (PSS 1 and PSS 2) and academic performance at T1 (Acad 1) and T2 (Acad 2) after controlling for effect of gender

				Gender		EI	
Variable	r	R^2^	Standard error of the estimate	β	*p* value	β	*p* value
PSS 1	0.333	0.104	5.38	–	–	−0.333	<0.001
PSS 2	0.240	0.046	5.59	–	–	−0.240	0.028
Acad 1	0.294	0.064	12.25	−0.234	0.032	0.153	0.158
Acad 2	0.268	0.049	21.79	−0.240	0.029	0.095	0.382

**Table 5:** Comparison of emotional intelligence score (EI), and academic performance (Acad) of upper (Q4) and lower quartile (Q1) of participants categorized using percieved stress score at T1 (PSS1) and T2 (PSS2)

	Quartile category	EI	Acad
T1	Q1, PSS <14, *n* = 29	125.86 ± 9.12	38.7931 ± 12.00410
	Q4, PSS >23, *n* = 34	116.82 ± 12.50	33.1471 ± 14.39304
	*p* value	0.002*	0.10
T2	Q1, PSS <14, *n* = 36	123.94 ± 10.89	60.47 ± 20.56
	Q2, PSS >22, *n* = 37	116.00 ± 15.00	54.08 ± 24.79
	*p* value	0.012*	0.235

*Statistically significant

PSS score was also regressed stepwise on EQ and gender. PSS score was predicted by EI scores at both T1 (*r* = 0.333, β = −0.333, *p* < 0.001) and T2 (*r* = 0.240, β = − 0.240, *p* = 0.028). Results are provided in Table 4. Root mean square errors (RMSE) were almost same for both training and test data set at T1 as well as T2 with the derived equation. RMSE at T1 was 6.74 on test data set and 5.34 on training set. RMSE at T2 was 5.81 on test data set and 5.51 on training data set.

## Discussion

In the present study, we found self-report emotional intelligence was a significant negative predictor of perceived stress over time. This finding is in line with previous study which reported negative association between self-report emotional intelligence and perceived stress [16] in undergraduate medical students but did not determine the direction of relation. This study, on the other hand, validates this predictive ability by student level cross validation at two different times.

There was no relationship between perceived stress and academic performance in the present study. This result is in contradiction to many studies reporting negative association between them [25]. This may be explained based on the fact that PSS scores give no information whether the perceived stress is acting as performance enhancer (eustress) or depreciator (distress) which have significant impact on the performance [26] mediated by exam self-efficacy [27] and motivation [28]. This is supported by another study that found no association between perceived stress and academic performance when controlled for pre-medical-school performance [5], which has been found to be affected by students’ self-efficacy and motivation [29].

Alternatively, even though, we had rendered the questionnaires to the students one week before the examination to infer the stress perceived by students a month prior to exam stressor, we are not certain whether exam stressor actually had a month long effect on perceived stress. It is also supported by the surprising fact surfaced in the results that perceived stress significantly decreased in males despite their second exam being a pre university exam. Had we taken perceived stress scores at a time unrelated to examination and compared the stress levels with and without examination, we would have been able to infer the results more appropriately.

There was no relationship inferred between emotional intelligence and academic performance in the present study. It is consistent with previous reports where self-report measures have been shown to have no effect on end of year marks [15], though ability based measure of EI has been shown to have a positive impact on end of year marks and cumulative grades [30]. Trait emotional intelligence correlates with personality [31] and its effect on performance may vary depending on the emotional intelligence relevant content of the subject [32]. A recent systematic review also drew the same conclusion [33].

To the best of our knowledge, this is one of the few studies, which validates the predictive ability of emotional intelligence on perceived stress in undergraduate medical students over time. Even if the percentage variability in perceived stress appears to be small, the process of validation gives evidence to the calls of training the medical students for increasing their emotional intelligence to deal with stress and improve their well-being. Research on training young adults for increasing their emotional intelligence has already shown promising results as after training they are better able to comprehend and manage their emotions and show significant improvement in their trait emotional intelligence [34]. However, additional research should explore the effect of unmeasured factors, not included in this study, like general intelligence, number and quality of stressful factors and coping strategies on perceived stress by means of structure equation modeling to understand the relationship in a better way.

Nonetheless, to consider the implications of the present study, certain limitations need to be warranted. First, as students participated voluntarily in the study, there might be a selection bias. Low response rate at T2 can be a source of bias as more stressed students at pre-university exams might have chosen to drop out. Second, emotional intelligence of participants of present study might have changed over time, though it appears unlikely given the short interval between administrations of the questionnaires and as self-report emotional intelligence does not change over time [17, 35]. Third, we have taken marks of only one subject and only cognitive domain for assessing academic performance. It was perhaps unsurprising that trait emotional intelligence did not significantly correlate with academic performance based on written tasks. Hence, in future studies, measures of performance with higher emotional content, such as clinical communication, should be considered [36].

## Conclusion

Though trait emotional intelligence has no effect on academic performance but it predicts stress perceived by 1st year medical students. It also suggests that medical colleges may adopt the strategies to develop emotional intelligence of medical students to improve their well-being.
